# Body wall structure in the starfish *Asterias rubens*

**DOI:** 10.1111/joa.12646

**Published:** 2017-07-16

**Authors:** Liisa M. Blowes, Michaela Egertová, Yankai Liu, Graham R. Davis, Nick J. Terrill, Himadri S. Gupta, Maurice R. Elphick

**Affiliations:** ^1^ School of Biological & Chemical Sciences Queen Mary University of London London UK; ^2^ School of Engineering & Materials Science Queen Mary University of London London UK; ^3^ Institute of Dentistry Barts and The London School of Medicine and Dentistry Queen Mary University of London London UK; ^4^ Diamond Light Source Ltd Didcot Oxfordshire UK

**Keywords:** body wall, collagen, echinoderm, ossicle, scanning synchrotron small‐angle X‐ray diffraction, starfish, X‐ray microtomography

## Abstract

The body wall of starfish is composed of magnesium calcite ossicles connected by collagenous tissue and muscles and it exhibits remarkable variability in stiffness, which is attributed to the mechanical mutability of the collagenous component. Using the common European starfish *Asterias rubens* as an experimental animal, here we have employed a variety of techniques to gain new insights into the structure of the starfish body wall. The structure and organisation of muscular and collagenous components of the body wall were analysed using trichrome staining. The muscle system comprises interossicular muscles as well as muscle strands that connect ossicles with the circular muscle layer of the coelomic lining. The collagenous tissue surrounding the ossicle network contains collagen fibres that form loop‐shaped straps that wrap around calcite struts near to the surface of ossicles. The 3D architecture of the calcareous endoskeleton was visualised for the first time using X‐ray microtomography, revealing the shapes and interactions of different ossicle types. Furthermore, analysis of the anatomical organisation of the ossicles indicates how changes in body shape may be achieved by local contraction/relaxation of interossicular muscles. Scanning synchrotron small‐angle X‐ray diffraction (SAXD) scans of the starfish aboral body wall and ambulacrum were used to study the collagenous tissue component at the fibrillar level. Collagen fibrils in aboral body wall were found to exhibit variable degrees of alignment, with high levels of alignment probably corresponding to regions where collagenous tissue is under tension. Collagen fibrils in the ambulacrum had a uniformly low degree of orientation, attributed to macrocrimp of the fibrils and the presence of slanted as well as horizontal fibrils connecting antimeric ambulacral ossicles. Body wall collagen fibril D‐period lengths were similar to previously reported mammalian D‐periods, but were significantly different between the aboral and ambulacral samples. The overlap/D‐period length ratio within fibrils was higher than reported for mammalian tissues. Collectively, the data reported here provide new insights into the anatomy of the body wall in *A. rubens* and a foundation for further studies investigating the structural basis of the mechanical properties of echinoderm body wall tissue composites.

## Introduction

The phylum Echinodermata comprises five extant classes: the Asteroidea (starfish), Ophiuroidea (brittle stars), Echinoidea (sea urchins and sand dollars), Holothuroidea (sea cucumbers) and Crinoidea (sea lilies and featherstars) (Brusca et al. 2016). A characteristic feature of echinoderms is their endoskeleton, which comprises magnesium calcite ossicles, or plates, together with varying proportions of associated collagenous and muscular tissue. At one end of the spectrum are sea urchins where the body wall comprises fused calcite plates that form a rigid and inflexible endoskeleton (Birenheide et al. 1996; Takemae & Motokawa, 2005; Ribeiro et al. 2011, 2012b; Motokawa & Fuchigami, 2015). At the other end of the spectrum are sea cucumbers, where the body wall largely comprises flexible collagenous tissue that contains only a scattering of tiny calcite spicules (Motokawa, 1984; Byrne, 2001). Intermediate compositions are found in asteroids, ophiuroids and crinoids, where the body wall comprises calcite ossicles or plates that are interlinked by muscles and collagenous ligaments, which confer skeletal flexibility (Eylers, 1976; Motokawa, 1984, 2011; O'Neill, 1989; Birenheide & Motokawa, 1996). Furthermore, there is variability between starfish species in the proportions of the calcite ossicles, muscle and collagenous tissue in the body wall (Heddle, 1967; Motokawa, 2011).

The first detailed investigation of the structure and mechanical properties of the starfish body wall was a study by Eylers (1976) on *Asterias forbesi* Desor, 1848 (Forcipulatida). The structure of the ray skeleton was investigated by analysis of preparations in which the majority of the soft tissue had been dissolved to reveal the architecture of the network of calcite ossicles. Different ossicle types were identified in different regions of the ray, including the ambulacral and adambulacral ossicles on the oral side and the marginal, reticular and carinal ossicles in the aboral body wall. Subsequently, O'Neill (1989) analysed the spatial organisation of ossicles in the aboral body wall of the starfish *Echinaster spinulosus* Verrill, 1869 (Spinulosida) and identified two ossicle types – oblate disks and ellipsoid bars, which form a reticular lattice of hexagonal rings.

The ultrastructure of ossicles in the starfish *Pisaster giganteus* Stimpson, 1857 (Forcipulatida) has been investigated using powder X‐ray diffraction, infrared spectroscopy, elemental analysis and scanning electron microscopy (SEM) (Martina et al. 2005; Gayathri et al. 2007). This revealed that starfish ossicles are composed of single crystals of macroporous magnesium‐rich calcite (stereom), similar to the mineralised skeletal elements of echinoids and some ophiuroids (Raz et al. 2000; Aizenberg et al. 2001; Addadi et al. 2003; Long et al. 2014). X‐ray microtomography (Micro‐CT) has also been employed to analyse the 3D structure of echinoderm endoskeletons, but the use of this technique has largely focused on fossil specimens (Dominguez et al. 2002; Zamora et al. 2012; Rahman et al. 2015); only more recently have extant species been examined (Ziegler et al. 2010, 2014).

The presence of muscles and connective (collagenous) tissue connecting, in parallel, adjacent ossicles of the starfish body wall has been revealed by histochemical staining of thin sections (Hamann, 1885; Smith, 1936; Eylers, 1976; O'Neill, 1989). O'Neill (1989) described the collagenous component of the body wall as a ‘loosely woven, three‐dimensional fabric in which some of the fibres were ‘felted’ (frayed together)’ and where interconnected fibres distribute load across the tissue and prevent delamination under strain. Besides the load‐bearing collagenous tissue component, O'Neill (1989) also described the interossicular (or ‘reticular’) muscles and muscle layers associated with the coelomic lining of the body wall.

Changes in posture of the starfish ray are mediated by the interossicular muscles (Heddle, 1967), whereas changes in the stiffness of the body wall are thought to be mediated by interossicular ‘catch’‐type collagenous tissue or mutable collagenous tissue (MCT; Motokawa, 1984; Wilkie, 2005). MCT is a characteristic of all echinoderms, with a unique ability to change stiffness rapidly under the control of the nervous system (Wilkie, 1996, 2002, 2005; Takemae & Motokawa, 2005). The mechanisms of echinoderm MCT are not fully understood. However, it has been proposed that the mutability is conferred not by changes in the mechanical properties of the collagen fibrils but by changes in the stiffness of the interfibrillar matrix (Cluzel et al. 2001; Wilkie, 2005; Ribeiro et al. 2012a). Recently, this mechanism was demonstrated at the nanoscale using sea cucumber dermis as an experimental system (Mo et al. 2016). Furthermore, proteins that may mediate changes in interfibrillar stiffness have also been identified (Motokawa, 1984; Trotter et al. 1996, 1999; Birenheide et al. 1998; Koob et al. 1999; Tipper et al. 2003; Tamori et al. 2006; Yamada et al. 2010).

Hitherto, the techniques used to study the soft tissue phase (collagenous and muscle components) of the starfish body wall have been predominantly histological (e.g. trichrome staining) (Smith, 1936; Heddle, 1967; Eylers, 1976; O'Neill, 1989). However, scanning and transmission electron microscopy (SEM and TEM) have also been used to analyse the ultrastructure of fixed echinoderm collagen tissue or extracted fibrils (Thurmond & Trotter, 1994; Trotter et al. 1994; Ribeiro et al. 2011; Barbaglio et al. 2015). Fixation and extraction can induce changes in the native structure of the collagen fibrils and therefore alternative methods without use of fixatives are also needed. Microfocus scanning synchrotron small‐angle X‐ray diffraction (SAXD) is one such technique that has proven to be a powerful and informative approach for analysis of collagenous tissue structure at the nanoscale (Sasaki & Odajima, 1996a; Puxkandl et al. 2002; Fratzl, 2008; Krauss et al. 2009; Yang et al. 2015). Collagenous tissues are amenable to SAXD because collagen molecules are arranged in a highly ordered manner within fibrils, resulting in alternating electron‐dense and electron‐light regions along the long axis of the fibrils. The combined width of adjacent electron‐dense and electron‐light regions is known as the D‐period, which is ~ 65–67 nm in mammals, depending on factors such as tissue type and level of hydration (Fratzl, 2008). When collagenous tissue is irradiated with X‐rays, the two‐phase periodic arrangement of electron densities leads to diffraction peaks in the meridional direction, enabling the D‐period and fibril orientation to be determined. As long as exposure times to X‐rays are not too long (from seconds down to milliseconds depending on the brilliance of the X‐ray source), the tissue can be examined in its native state (Jeffries et al. 2015). In this way, SAXD has been used successfully to analyse the structural and mechanical properties of, for example, mammalian tendons, bone and antlers (Gupta et al. 2006; Zimmermann et al. 2011; Yang et al. 2015; Karunaratne et al. 2016). Furthermore, it was use of SAXD that recently enabled interfibrillar stiffening of echinoderm MCT to be demonstrated at the nanoscale, using sea cucumber dermis as an experimental system (Mo et al. 2016).

To obtain new insights into relationships between body wall structure and function in starfish, here we have used histology, micro‐CT and SAXD to perform a detailed analysis of the structural properties of the body wall in the common European starfish *Asterias rubens* Linnaeus, 1758 (Forcipulatida).

## Materials and methods

### Animals

Starfish (*A. rubens*) used in this study were collected at low tide from Foreness Point near Margate in Kent. Animals were transported in seawater to Queen Mary University of London where they were then maintained in an aquarium with circulating artificial seawater at ~ 11 °C, corresponding to the natural temperature of sea water for this species. Specimens ranging in diameter from 3 to 7 cm were used for studies reported in this paper. Animals were fed periodically with the mussel *Mytilus edulis* Linnaeus, 1758 (Mytiloida).

### Masson's trichrome staining

To enable analysis of the histological composition of the body wall in *A. rubens*, Masson's trichrome staining was used; this differentiates collagenous (blue) from non‐collagenous (red) tissues. Intact animals or severed rays were fixed for 24 h in Bouin's fixative, which comprised 75 mL of saturated picric acid in seawater, 25 mL 40% formaldehyde solution and 5 mL glacial acetic acid. Following fixation, specimens were decalcified at 4 °C for 2–5 days in a freshly prepared solution of 1% ascorbic acid in 0.15 m sodium chloride. Prior to fixation, some specimens were narcotized in seawater containing 3.5% added MgCl_2_, which causes muscle relaxation (Mayer, 1909). This was done to reduce the tissue damage caused during fixation and tissue processing that was observed in untreated specimens. Following fixation and decalcification, starfish rays were dehydrated through an ethanol series, immersed in xylene and then embedded in paraffin wax. Serial transverse sections of rays were prepared using a Leica RM2145 microtome and mounted on chrome alum gelatin‐coated slides. Following dewaxing in xylene and rehydration, slides were stained using Masson's trichrome method. Sections were rehydrated and then placed in Weigert's haematoxylin solution for 10 min and then washed in running tap water for 10 min at room temperature. After rinsing with distilled water, slides were placed in Biebrich scarlet‐acid fuchsin solution for 15 min and then washed in distilled water. Differentiation of staining was achieved by placing the slides in phosphomolybdic‐phosphotungstic acid solution until collagenous tissue no longer appeared red (normally 10–15 min). Slides were then transferred into aniline blue solution for 5–10 min, followed by washes (3 × 1 min) in distilled water and then a 1–5 min differentiation step in 1% acetic acid. After washing in distilled water, slides were quickly dehydrated in 95% (1 × 10 s) and 100% ethanol (3 × 10 s), cleared in xylene and then mounted using DPX, a resin‐based mounting medium (Thermo Fisher Scientific Inc.).

### X‐ray microtomography

Micro‐CT was used to enable analysis of the structure and organization of the complex network of calcareous ossicles that form the endoskeleton of the body wall in *A. rubens*. Starfish were fixed (overnight at 4 °C) with a 4% solution of paraformaldehyde in artificial seawater and then washed in phosphate‐buffered saline (PBS; pH 7.4). The rays were cut off and embedded in 4% low melting point agarose dissolved in PBS, contained within 5 mL polystyrene tubes, which were then stored at 4 °C. The complete tubes were scanned on the custom‐designed MuCAT 2 micro‐CT scanner at Queen Mary University of London. This uses a high dynamic range CCD camera in time‐delay integration mode to achieve accurate, high contrast tomographic images (Davis et al. 2013). The accelerating voltage was set to 40 kV with a tube current of 405 μA. The voxel size was set to 15 μm; 1201 projections were recorded in 19 h (the long scan time is required to obtain a high contrast ratio and two complete scans were required to cover the ~ 20 mm length of the rays). Immediately following the scan, a multi‐element calibration carousel (Evershed et al. 2012) was scanned to characterise the X‐ray spectrum. As direct measurement of the X‐ray spectrum is almost impossible (with the degree of accuracy required), a modelled estimate of the spectrum was made. Attenuation measurements from the carousel were used to optimize the model to create a good working representation of the spectrum. With *a priori* knowledge of at least the most absorbing components of the specimen, a correction curve can be created that maps the projections derived from polychromatic radiation to an estimate of those that would be derived from monochromatic radiation. This both corrects for beam‐hardening errors and allows measurements of mineral concentration to be made from the reconstructed image. Although high magnesium calcite is likely the appropriate mineral in the case of starfish ossicles (Gayathri et al. 2007), as we did not know the exact chemical composition and were not interested in absolute mineral concentration measurements, hydroxyapatite mineral was used to create the correction curve. Reconstructed volume images were viewed from different orientations using in‐house tomview software and rendered using the Drishti volume rendering package to produce 3D views (Limaye, 2012).

### *In situ* synchrotron small‐angle X‐ray diffraction

#### SAXD experiments on starfish aboral body wall and ambulacrum strips

**Figure 1:**
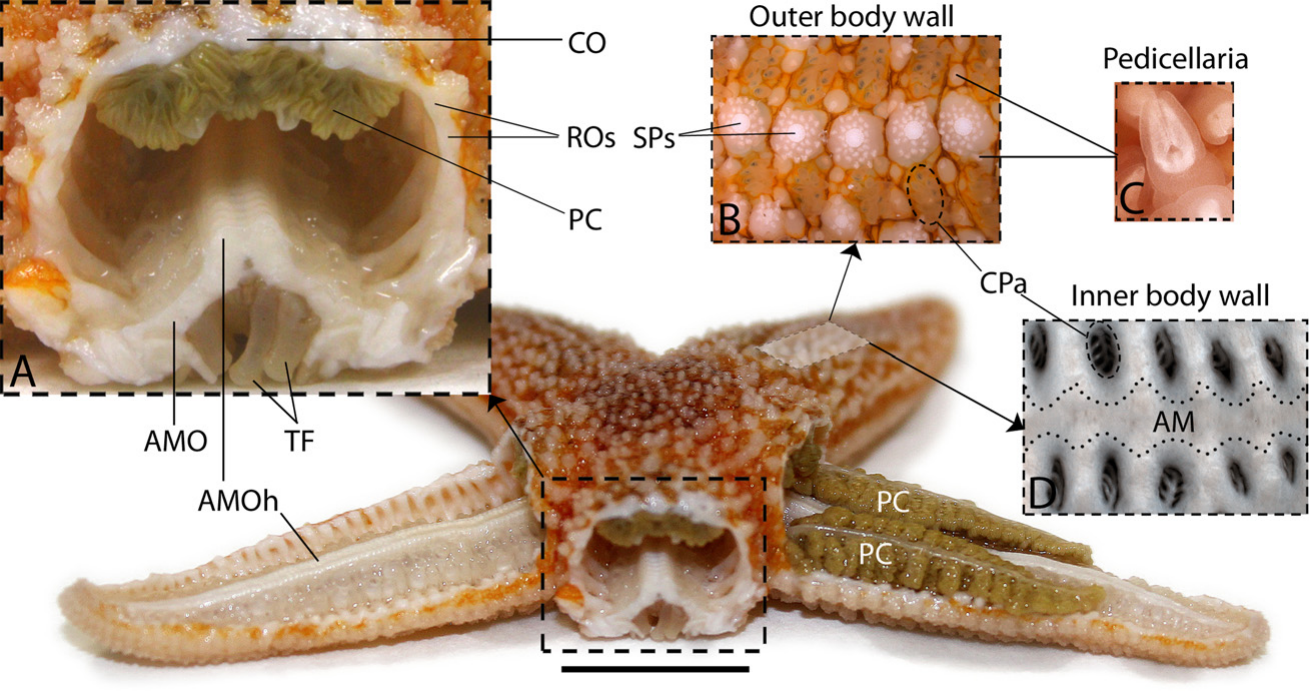
General anatomy of starfish *Asterias rubens*. The main image shows a specimen of the starfish *A. rubens* with one of the rays transversely dissected and with the aboral body wall of two other rays removed to reveal the digestive glands (pyloric caeca, PC) and the ridge of ambulacral ossicle heads (AMOh). Inset (A) shows a close‐up of the transverse cross‐section of the ray with ambulacral ossicles (AMO) and tube feet (TF) on the oral side of the cross‐section and pyloric caeca attached to the inner side of the aboral body wall. The positions of the apical carinal ossicle (CO) and lateral reticular ossicles (ROs) are labelled. Inset (B) shows the outer surface of the aboral body wall centred on the mid‐line of the arm showing the positions of spines (SPs) that are located over the row of carinal ossicles and clusters of papulae (CPa). Inset (C) shows a close‐up of a pedicellaria, a pincer‐shaped defensive organ. Inset (D) shows a close‐up of the inner surface of the aboral body wall with voids in the body wall that are overlain by clusters of papullae (CPa) on the outer body wall surface (see inset C). The position of the longitudinally orientated apical muscle (AM) is outlined. Scale bar (on main figure): 1 cm.

**Figure 2:**
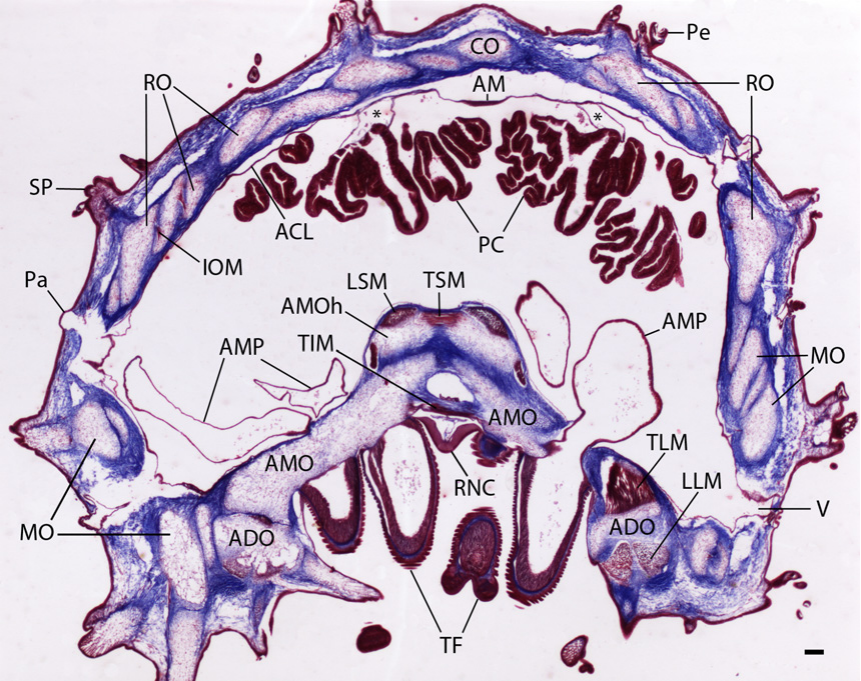
Trichrome‐stained transverse section of a decalcified ray from *Asterias rubens*. The voids formed by decalcification of the body wall ossicles show the positions and shapes of the different ossicle types: the ambulacral ossicles (AMO), the adambulacral ossicles (ADO), the marginal ossicles (MO), the reticular ossicles (RO) and the carinal ossicle (CO). The red speckling within the ossicle voids are stained cells, which are located in pores between the calcite struts of the ossicle stereom *in vivo*. Surrounding the ossicle network can be seen a dense meshwork of collagenous tissue (blue), which forms the bulk of the soft tissue in the body wall. The interossicular muscles that link adjacent ossicles can also be seen. These are most prominent adorally, where there are large muscles that link adjacent ambulacral ossicles (longitudinal supra‐ambulacral muscle, LSM; transverse infra‐ambulacral muscle, TIM; transverse supra‐ambulacral muscle, TSM) or that link ambulacral ossiscles with adambulacral ossicles (inner and outer transverse lateral muscles; TLM; longitudinal lateral muscles, LLM). The smaller interossicular muscles (IOM) linking ossicles of the aboral skeleton are also evident but these are seen more clearly at higher magnification (see Fig. 3). Occupying the coelomic space internal to the body wall can be seen the prominent pair of digestive glands (pyloric caeca, PC), which are connected via mesenteries (*) to the aboral coelomic lining (ACL) of the body wall. The coelomic lining is detached from the body wall in this stained section, which is an artifact probably caused by shrinkage of the body wall dermis during tissue processing. Note that the aboral lining of the coelom is thicker in the midline position due to the presence of the longitudinally oriented apical muscle (AM), which causes aboral flexion of the ray when it contracts *in vivo*. The prominent V‐shaped radial nerve cord (RNC) can be seen between the two rows of tube feet podia (TF), which are connected to the intracoelomic bulb‐shaped ampullae (AMP) by tubular connections that run between adjacent ambulacral ossicles (as seen here on the right side). Note also other appendages that are associated with the external body wall surface, including spines (SP), pedicellariae (Pe) and papulae (Pa) that overlay voids (V) between the ossicles forming the aboral body wall skeleton. Scale bar: 150 μm.

Experiments were performed using the beamline I22 synchrotron light source at Diamond Light Source (DLS; Oxfordshire, UK). Live specimens of *A. rubens* were transported in a portable tank to DLS, where they were maintained in cool seawater until immediately prior to dissection of body wall samples from two individuals. Strips of the midline aboral body wall of rays were prepared using a pair of parallel‐blades that were separated by a 6‐mm gap. Strips of the ambulacrum on the oral side of rays were prepared by cutting between the rows of ambulacral and adambulacral ossicles on both sides of the ray (see Figs 1 and 2). The SAXS scans of the ambulacrum and aboral body wall were carried out at different times separated by wide‐angle X‐ray diffraction measurements on other samples at the same beamline.

For static scanning SAXD tests, the strips were placed in a 50 × 50 mm plastic clamp, with a hole of 35 × 23 mm in the centre. The hole was covered with Ultralene film (Spex SamplePrep, Metuchen, NJ, USA), which has minimal X‐ray scattering in the small‐angle regime. Artificial seawater (ASW, prepared according to the manufacturer's instructions using Tropic Marin^®^ Sea Salt) was added inbetween the two layers of film where the sample was held in order to provide a physiological environment for the samples. The edges of the body wall samples were marked with X‐ray absorbent lead tape to aid detection of the sample centre when scanning with the X‐ray beam.

The sample holder was clamped on a 2‐axis motorized stage and a synchrotron X‐ray beam with a wavelength of 0.8857Å and beam diameter 15 μm was used to acquire the SAXD spectra. Spectra were collected using a silicon hybrid pixel PILATUS 2M detector system (Kraft, 2010). The sample‐detector distance was 1008.2 mm for aboral body wall samples and 847.2 mm for ambulacral samples, calculated based on a measurement of a calibration standard consisting of silver behenate. The detector had a 2D resolution of 1475 × 1678 pixels with pixel dimensions of 172 × 172 μm^2^.

The centre of the sample on the plastic frame was first located by carrying out a 2D X‐ray absorption (radiography) scan of the sample across the X‐ray beam with a photodiode placed downstream of the sample. The strong X‐ray absorption by the lead markers enabled location of regions of interest on the specimen. 2D SAXD area‐scans, with 40 and 100 μm x‐ and y‐step distances used for ambulacral and aboral body wall, respectively, were acquired in the regions of interest using software for customized control of motorized stage movement and detector SAXD pattern acquisition, available at the beamline via the GDA open source framework (http://www.opengda.org/). Different spatial resolutions were used for the two body regions because of their intrinsically different size. To avoid radiation damage by repeated exposure of the same tissue location to the beam, the 2‐axis motorized stage was translated by an offset of 30 μm (~ 2× beam diameter) in the x‐direction between carrying out the 2D diode and SAXD scan grids.

#### SAXD data analysis

##### Identification of diffraction peaks arising from collagen D‐period

2D SAXD spectra were first corrected for air and background scattering by the empty cell, using photodiode measurements of the transmission of the sample and the empty cell, as is standard practice in SAXD data acquisition (Pabisch et al. 2013). The corrected 2D spectra were initially averaged azimuthally across a 0–360° sector (with a wavevector range from 0.2 to 0.9 nm^−1^) to identify points in the 2D scan where SAXD peaks arising from collagen are present (subsequent more detailed analysis of fibril orientation, D‐period and peak intensity is described below). The Bragg peaks arising from the 65–68 nm axial D‐period of the collagen fibrils were identified qualitatively from expected positions in the spectrum, with the *n* = 5th order (at q ~ 0.46 nm^−1^) being the clearest peak.

#### Determination of collagen fibril orientation

To determine collagen fibril orientation in body wall samples, the angular distribution of the SAXD intensity of the 5th order reflection I_5_(χ) was used. I_5_(χ) was calculated by averaging the intensity radially in a narrow band around the peak position of the 5th order reflection, i.e. over the wavevector range 0.45–0.55 nm^−1^. The intensity profile was calculated for the full angular range in χ from 0 to 360°. To correct for diffuse background scattering, the average of the intensity in two narrow rings (just outside the ring of 0.45–0.55 nm^−1^ containing the *n* = 5 reflection) was subtracted from the ring centred on the peak position, following the same procedure as described for bone collagen in Karunaratne et al. (2016). The corrected intensity I_5_(χ) exhibited two peaks separated by 180° and hence was fitted to the sum of two Gaussians separated by 180° using the gnuplot graphics and fitting program (www.gnuplot.info). Peak amplitude, peak width (Δχ_0_) and peak position (χ_0_) were determined from the fit. χ_0_ determines mean fibril orientation and the reciprocal of peak width $\left(\frac{1}{\Delta \chi_{0}}\right)$ was used as a measure of the degree of fibrillar orientation around the mean fibril orientation. Lower values of $\left(\frac{1}{\Delta \chi_{0}}\right)$ imply a more random dispersion of fibril directions, whereas higher values imply a more aligned arrangement.

Following identification of fibril direction, the D‐period for each 2D pattern was calculated from azimuthally averaged intensity profiles denoted I_q_, with q the radial wavevector; the total intensity of a particular peak order (the integrated intensity under the Bragg peak) will be denoted with a subscript, for example the 5th order peak intensity will be denoted as I_q5_. Specifically, the 2D spectra were azimuthally averaged again in a pie‐shaped sector centred at the mean fibril orientation, and covering 20° on either sides of χ_0_. This procedure ensures that the signal is not attenuated by including angular sectors, which are far from the principal fibril directions and have no scattering intensity from the collagen meridional peaks. The most clearly visible orders were the 5th (*n* = 5, I_q5_) and 8th (*n* = 8, I_q8_), with the 5th order having the strongest intensity.

### Determination of fibril D‐period and peak intensity

As the 5th order Bragg peak from the collagen D‐period was the most prominent, it was selected for fitting a Gaussian peak function with a linear baseline (background) term for each SAXD spectrum. The peak position q_5_, peak amplitude A_5_, and peak width‐at‐half‐maximum w_mq5_ were determined from the fits, as reported previously in Mo et al. (2016). The meridional peak width is influenced by instrumental broadening of the peak due to the finite resolution of the detector. The instrumental broadening of the peak can be calculated from diffraction patterns of collagen fibrils with highly crystalline organization, such as in dried rat tail tendon or chicken collagen (Krauss et al. 2009). The corrected full‐width‐at‐half‐maximum (FWHM) was calculated, as described in Krauss et al. (2009), according to the formula $w_{q5}=\sqrt{w_{mq5}^{2}-w_{crys}^{2}}$ where *w*_*q5*_ is the corrected FWHM, *w*_*mq5*_ is the measured FWHM of the sample of interest, and *w*_*crys*_ is the FWHM of the sample with near crystalline organization (here chicken collagen was used). The peak width w_q5_ represents the variability of q_5_ at each scan point. As the D‐period length is calculated using the formula $D=5\times \frac{2\pi }{q5}$ (as calculated similarly for the 3rd order meridional reflection in bone collagen: e.g. Karunaratne et al. 2012), w_q5_ is also a measure of the variability of D‐period length at any scan point. The range of D‐values at each scan point can therefore be derived from the above after determining the maximum and minimum q values q_5_ ± $\frac{1}{2}$w_q5_, respectively (the use of the half‐width as representing maximum and minimum is a convention choice). The azimuthal average of the intensity at the peak position (I_q5_ and I_q8_ referring to the intensities of the 5th and 8th order peaks, respectively) contains contributions from both the collagen phase and all the other components of the tissue. The diffraction peak intensity is from collagen (I_q5col_) and the background scattering is contributed by the mineral (I_q5min_) components of the tissue. Therefore to calculate I_q5col_, the background scattering (I_q5min_) was approximated as a linear fall‐off over the small q‐range around the peak and subtracted from I_q5_ to get I_q5col_. The linear background scattering was determined by calculating a linear regression over a combination of several points to the left and right of the peak.

As not all scan points were on collagen‐containing regions, a filtering process was used to identify SAXS patterns with sufficient collagen for analysis, based on the multi‐step procedure outlined above. Points with little scattering intensity are expected to give artefactual results for fitting, such as very small or negative peak width, low peak amplitude or very large errors in the fit. After visual inspection of the Bragg peaks of some selected patterns, integrated profiles and fit results, only scan points satisfying the following empirical criteria were determined to have substantial amounts of collagen and were included in the data analysis: (i) positive Δχ_0_; (ii) w_q5_ > 0.0085 nm^−1^ [very noisy and low intensity I(q) tended to have very small w_q5_ < 0.0001 nm^−1^] and (iii) sum of errors (the sum of % error of the peak fit parameters q_05_, A_5_ and w_q5_) < 17%. This corresponded to ~ 22% of all the scan points.

### Determination of the overlap: D‐period ratio in collagen fibrils

Structural information on the intrafibrillar arrangement of tropocollagen molecules can be obtained from the differences in peak intensities between the different orders of the SAXD spectrum. Specifically, assuming the gap zone inside the fibril to have a length G and the overlap zone to have a length O and a uniform electron density in each zone, with O + G = D, then it can be shown (Sasaki & Odajima, 1996a) that the ratio of the peak intensities of the n_1_th peak to that of the n_2_th peak is:

$$\frac{I_{n_{1}}}{I_{n_{2}}}={\left(\frac{n_{2}}{n_{1}}\right)}^{2}\left(\frac{sin\left(\pi n_{1}\frac{O}{D}\right)}{sin\left(\pi n_{2}\frac{O}{D}\right)}\right)$$

The two most prominent peaks in the radial profile I_q_ were the 8th and 5th order. Hence, from the ratio of the intensities of the n_1_ = 8th order peak to the n_2_ = 5th order peak (I_q8_/I_q5_) the ratio O/D was determined by numerically solving the above equation for O/D, given an experimentally measured intensity ratio. It is noted that the assumption of a constant electron density is likely not strictly accurate, as has been found in vertebrate collagen types (Antipova & Orgel, 2010), but in the absence of detailed information on the amino acid sequences and structure of collagen in starfish body wall, we use it as an approximation.

## Results

To facilitate interpretation of data obtained using histological staining, micro‐CT and SAXD (see below), Fig. 1 shows the anatomy of a dissected specimen of *A. rubens*.

### Histology of the body wall of *A. rubens*

**Figure 3:**
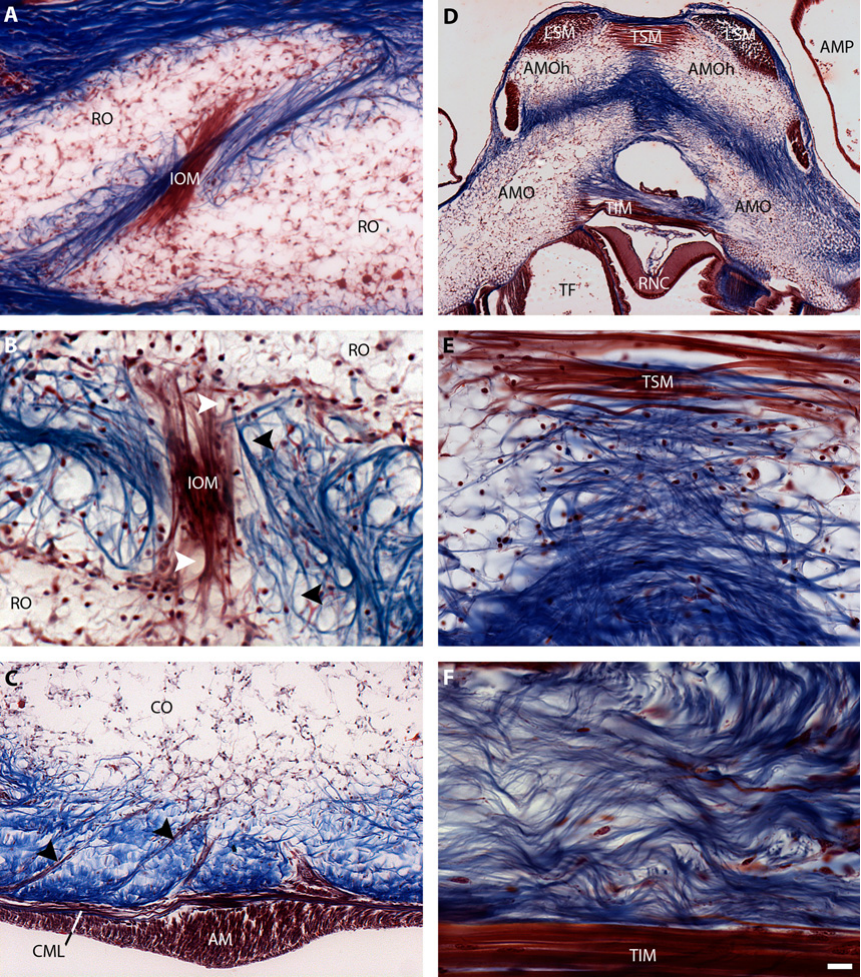
Trichrome stained sections of starfish body wall showing ossicles and associated muscles and collagenous tissue. (A) Adjacent reticular ossicles (RO) are linked by an interossicular muscle (IOM) and are embedded within a collagenous tissue meshwork (blue). The calcareous struts of the ossicles appear as voids, due to decalcification of the tissue, and the cellular stroma appears red. (B) High magnification image of adjacent reticular ossicles showing how muscle fibres of an interossicular muscle (IOM) insert between and around strut voids near the surface of each ossicle (white arrows). The wrapping of collagen fibres (blue) around ossicle strut voids (black arrows) can be clearly seen in this image. (C) Muscle strands (arrows) derived from the circular muscle layer (CML) above the apical muscle (AM; longitudinal muscle) extend through the collagenous inner dermis (blue) and insert on the carinal ossicle (CO). (D) Ambulacral ossicle heads (AMOhs) are inter‐connected by transverse supra‐ambulacral muscle (TSM) and longitudinal supra‐ambulacral muscles (LSMs). The ambulacral ossicles (AMOs) are furthermore connected by transverse infra‐ambulacral muscle (TIM). Other abbreviations as in Fig. 2. (E). Collagen fibres (blue) below the transverse supra‐ambulacral muscle (TSM) connect antimeric ambulacral ossicle heads strapping around ossicle struts. (F) The collagen fibres (blue) above the transverse infra‐ambulacral muscle (TIM) have a predominantly transverse horizontal orientation with a macro‐crimp (wavy appearance). Scale bar (3F): 50 μm (A), 25 μm (B), 50 μm (C), 100 μm (D), 12.5 μm (E), 7.8 μm (F).

Figure 2 shows a trichrome‐stained section of a decalcified ray from *A. rubens*, with the calcite ossicles that are present *in vivo* appearing as voids because of the decalcification process. Here we focus our attention on the structure and organization of collagenous (blue) and non‐collagenous (red) tissues that surround and interlink the ossicles, which are shown at higher magnification in Fig. 3.

Adjacent ossicles in the body wall are interlinked by muscles and the sizes and shapes of these muscles vary between ossicle types. Examples of the small muscles that link ossicles in the aboral body wall are shown in Fig. 3, which shows interossicular muscles linking adjacent reticular ossicles (IOM, Fig. 3A,B). What these images also show is that muscle fibres of the interossicular muscles appear to be wrapped around voids that would be filled by calcite struts near the ossicle surface *in vivo*. In close association with the interossicular muscle is the collagenous tissue that surrounds and also interconnects adjacent ossicles. The collagenous tissue occupies the bulk of the space between ossicles and surrounding ossicles, with fibres forming a dense latticework (Fig. 3A,B). It can clearly be seen that the collagen fibres form loop‐shaped straps that wrap around the voids that would be filled by calcite struts near the ossicle surface *in vivo* (black arrow heads, Fig. 3C), Indeed, the entire surface of each ossicle is penetrated by collagen fibres that project into the ossicle.

The coelomic epithelium of the aboral body wall is underlain by two muscle layers: first, a layer of longitudinally orientated muscle, which is thickened along the midline to form what is known as the apical muscle AM, Fig. 3C, and secondly, a layer of circularly oriented muscle, from which are derived muscle strands that insert onto the inner surface of body wall ossicles (arrow heads, Fig. 3C).

The largest interossicular body wall muscles are the antagonistic pairs of muscles that link adjacent antimeric ambulacral ossicles (abductor *supra*‐ and adductor infra‐ambulacral muscles) and that link ambulacral and adambulacral ossicles (inner and outer lateral transverse muscles) (Figs 2 and 3D), which participate in enabling changes in ray posture (Heddle, 1967). The collagen fibres connecting the antimeric and longitudinally adjacent ambulacral ossicles are oriented predominantly horizontally (Fig. 3D), but slanted fibres connecting longitudinally overlapping ossicles between and within each side of the ambulacrum can also be observed in Fig. 3E. Crimping of collagen fibres in the interambulacral ligament located above the adductor infra‐ambulacral muscle can be seen in Fig. 3F.

### X‐ray microtomography reveals the 3D architecture of the starfish ray skeleton

**Figure 4:**
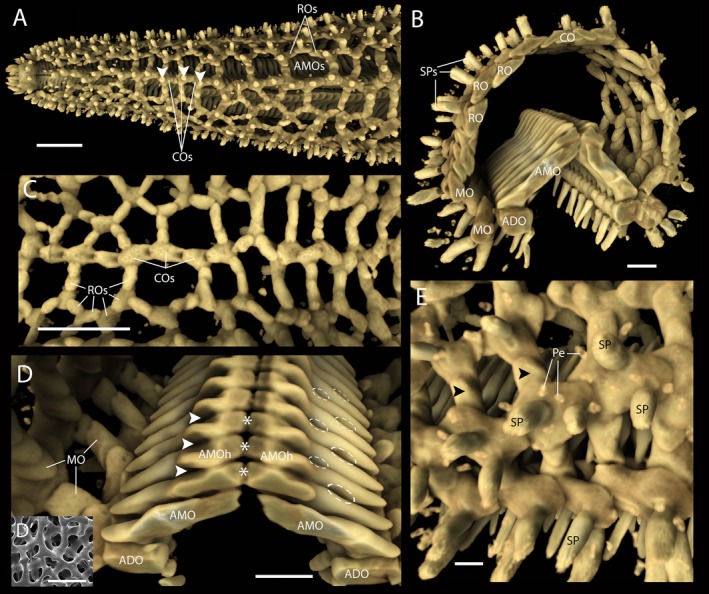
The ray skeleton of *Asterias rubens*, as revealed by X‐ray microtomography. (A) Low‐magnification overview of the ray skeleton from a top (aboral) view. Along the midline of the aboral skeleton can be seen the row of overlapping carinal ossicles (COs). Either side of the carinal ossicles are a loose meshwork of reticular ossicles (ROs) and through the gaps bounded by rings of reticular ossicles can be seen the two rows of ambulacral ossicles (AMOs) on the oral side of the ray. Note also the numerous spines located external to the ossicle network; three spines located above the carinal ossicles are labelled with arrowheads. (B) Transverse segment of a starfish ray showing the ambulacral skeleton formed by two rows of ambulacral ossicles (AMO), which are supported orally by the cuboid‐shaped adambulacral ossicles (ADO). Lateral to the adambulacral ossicles are the densely packed marginal ossicles (MO). The aboral region of the ray skeleton is formed by a loose meshwork of reticular ossicles (RO) and the single row of carinal ossicles (CO). Spines (SPs) can be seen on the body wall surface. (C) The aboral ray skeleton viewed from its underside, showing the overlapping row of carinal ossicles (COs) along the midline and the loose meshwork of reticular ossicles (ROs) on either side of the carinal ossicles. This image also illustrates how changes in orientation of the carinal and reticular ossicles, mediated *in vivo* by contraction/relaxation of interossicular muscles, affects skeletal structure. Thus, on the left hand side of the image ossicles form ring‐shaped structures, whereas on the right hand side of the image the ossicles form oblong‐shaped structures. (D) The ambulacral skeleton viewed at high magnification, looking towards the tip of the ray. The image shows how the slender and tightly packed ambulacral ossicles (AMO) are orientated at an angle, leaning away from the tip‐end of the ray. Furthermore, it can be seen that the aboral ‘head’ (AMOh) of each ambulacral ossicle overlaps an adjacent ossicle more proximal to the central disk. The large gaps between the adjacent ‘heads’ of ambulacral ossicles (arrowheads and asterisks) are occupied *in vivo* by longitudinally and transversely orientated interossicular muscles, respectively (which can also be seen in Figs 2 and 3). Dashed lines show where tubular connections of the tube feet and ampullae are located. (D') Scanning electron micrograph showing the calcite struts and pores of ambulacral ossicle stereom at high magnification. (E) External view of the marginal ossicles of the body wall. At this high magnification it can be seen that overlapping ossicles with appendages (spines, SP and pedicellariae, Pe) are arranged in longitudinally orientated rows and these ossicles are interlinked radially by smaller ossicles without appendages (arrowheads). Scale bars: (A,C) 2 mm; (B,D) 1 mm; (D’) 40 μm; (E) 500 μm.

Based on data obtained using micro‐CT to analyse the ray skeleton of *A. rubens*, here we present images that show for the first time the 3D architecture of the starfish skeleton in unprecedented detail (Fig. 4). A detailed description of the images shown in Fig. 4 is presented in the associated figure legend and therefore the focus here will be on a few key observations. What the images show most strikingly is the variety of shapes of the different ossicle types and the geometry of their interactions. Thus, the ambulacral ossicles are elongated, flattened and tightly packed with their aboral ‘head’ region orientated over the adjacent proximal ambulacral ossicle (Fig. 4D). These contrast with the more cuboid‐shaped adambulacral ossicles that provide basal support for the ambulacral ossicles (Fig. 4B). The marginal ossicles that form the lateral body wall region are more varied in shape and size but are quite tightly packed. Larger irregular‐shaped marginal ossicles form longitudinal overlapping rows of ossicles, which are connected radially by overlapping smaller, lozenge‐shaped ossicles (Fig. 4B,D). The reticular ossicles that form the aboral skeleton have a similar variety of ossicle shapes and sizes but these form a more open skeletal meshwork than that of the marginal ossicles (Fig. 4C). Lastly, the carinal ossicles form a single row of overlapping ossicles along the midline of the aboral ray skeleton, arranged like a fallen line of dominos toppled from the tip‐end of the ray (Fig. 4A,C).

Another feature that can be observed from the images shown in Fig. 4 are the shapes and sizes of what appear as unfilled gaps where the X‐ray attenuation is below the threshold set for the ‘hard tissue’ image rendering, and which is indistinguishable from the surrounding medium but would be filled with tissue *in vivo*. For example, linking the ‘head’ regions of the ambulacral ossicles are several muscles (Fig. 2) and the size and shapes of the spaces occupied by these muscles can be observed in Fig. 4D. Voids that would be filled by a narrow fluid‐filled tube linking the tube foot podium and ampulla can also be seen in Fig 4D, where they form two rows on each side of the ambulacrum in an alternating manner with a void proximal or distal to the head region of each pair of adjacent ambulacral ossicles. At the other end of the ambulacral ossicles, it can be seen that their bases insert in a groove formed by adjacent adambulacral ossicles (Fig. 4B–D), i.e. the ambulacral ossicles are not arranged directly above the adambulacral ossicles but are staggered so that these two interacting ossicle types alternate with respect to each other along the length of the ray.

### X‐ray transmission maps of aboral body wall and the ambulacrum reveal the positions of ossicles and spines

**Figure 5:**
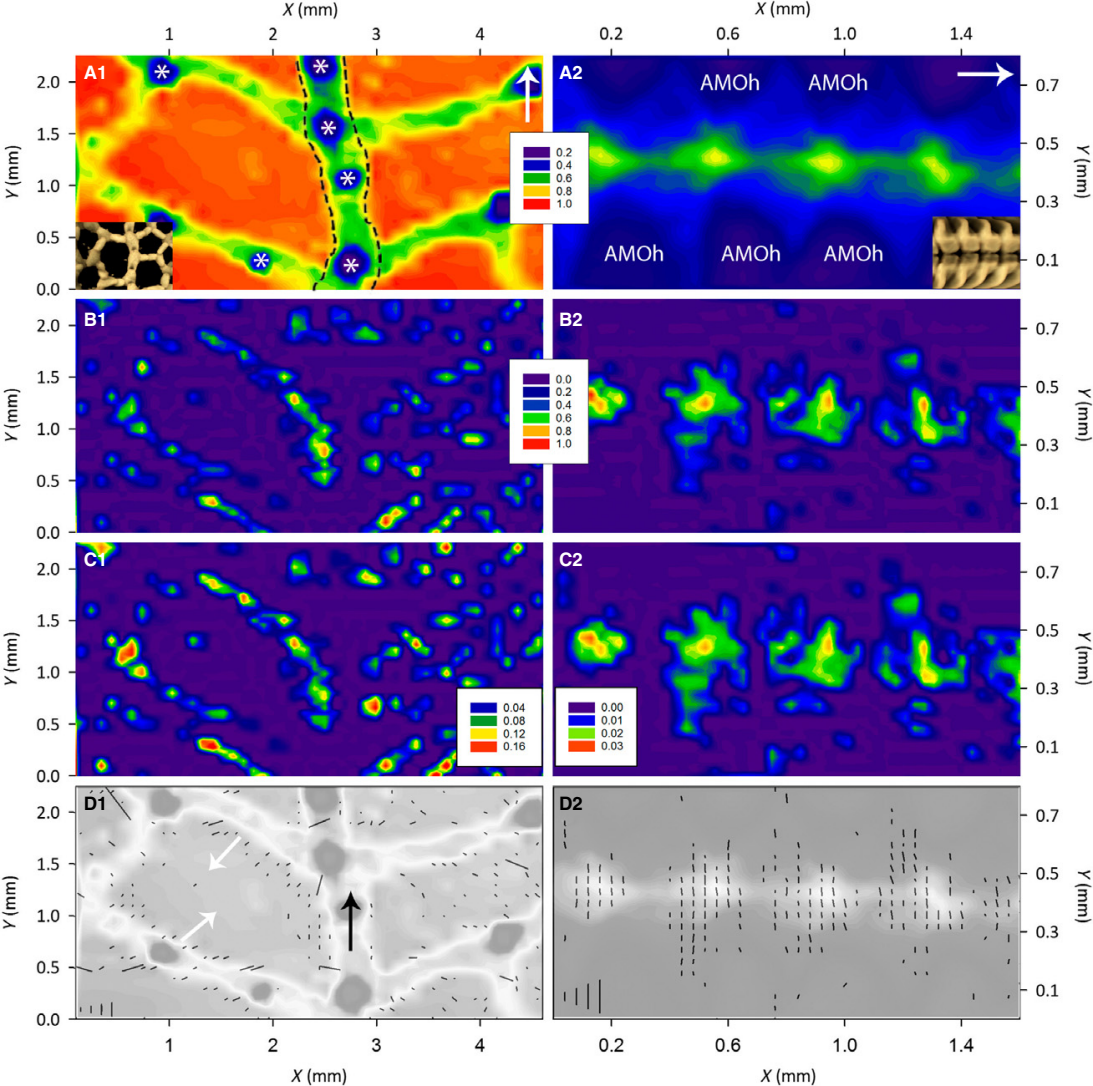
Transmission, I_q5col_; I_q5col_/I_q5min_ ratio and vector maps of *Asterias rubens* aboral body wall and ambulacrum. (A1,A2) X‐ray transmission maps of an aboral body wall (A1) and an ambulacrum sample (A2). The contour scale corresponds to darker regions as regions with higher density of the tissue (higher absorption). Areas of high density in blue (A1, *) indicate positions of spines. The insets are close‐ups of micro‐CT images of corresponding body locations. (B1,B2) Intensity of 5th order Bragg peaks mapped across the aboral (B1) and ambulacrum (B2) samples. Red corresponds to the highest amount of collagen. (C1,C2) Ratio of 5th order Bragg peak intensity from collagen fibrils (I_q5col_), to the intensity of diffuse SAX scattering (which arises mainly from mineral components of the tissue) (I_q5min_) mapped across the aboral (C1) and ambulacral samples (C2). (D1,D2) Vector and circle plots of collagen fibril structure overlapped with a transmission map of the aboral (D1) and ambulacral samples (D2). The orientations of the vectors are parallel to the orientation of the collagen fibrils. The length of the vector is inversely proportional to Δχ_0_. The degree of fibril orientation increases $\left(\frac{1}{\Delta \chi_{p}}\right)$ with the vector length. The scale vector lengths in the bottom left corner correspond to $\left(\frac{1}{\Delta \chi_{p}}\right)$ of 0.23, 0.46, 0.70 and 0.93 [Au] or Δχ_0_ of 5, 2.5, 1.67 and 1.25 degrees. It is noticable that all the vectors in D2 are shorter than the scale vector corresponding to Δχ_0_ of 5°, in comparison with 8% of vectors in D1 being longer than the scale vector for Δχ_0_ of 5°.

To interpret SAXD data with respect to anatomical organization of the starfish body wall, X‐ray transmission maps were obtained. Scanning X‐ray transmission images of the aboral body wall and ambulacrum revealed their inhomogeneous structure, with regional variation in X‐ray transmission and hence conversely X‐ray absorption (Fig. 5A1,A2; the longitudinal axis of the starfish ray is indicated by an arrow in these figures). Regions with high X‐ray absorption correspond with the location of the mineralized ossicles and associated appendages (e.g. spines, * in Fig. 5A1). Thus, the lattice‐like structure of the aboral endoskeleton that can be seen with micro‐CT (Fig. 4A,B) is also revealed in X‐ray transmission maps (Fig. 5A1). In Fig. 5A1 the outline of the row of carinal ossicles is indicated with dashed lines and it can be seen that the regions of highest X‐ray absorption (blue in Fig. 5A1) in the aboral body wall correspond with the positions of spines located on the outer surface of the body wall. In the ambulacrum, the overall level of X‐ray absorption is much higher than in the aboral body wall, consistent with the presence of the regular and densely packed rows of pairs of long ladle‐shaped ambulacral ossicles (Fig. 5A2, ambulacral ossicle head, AMOh). However, regions of lower X‐ray absorption are evident and these assist in identifying boundaries between adjacent ossicles. The regions of lowest X‐ray absorption are along the mid‐line of the ambulacrum, which is where the heads of the ambulacral ossicles align and are interlinked by muscles and collagenous tissue (Fig. 5A2).

### SAXD reveals the inhomogeneous distribution of collagen in the starfish body wall

The amount of collagen present in tissue is proportional to the intensity of the 5th order Bragg peak with the diffuse background scattering (I_q5min_) subtracted from it (I_q5col_, see Materials and methods). By mapping the I_q5col_ across the body wall samples (Fig. 5B1,B2), the areas with the highest concentrations of collagen (red) can be seen. Note, however, that the intensities shown in Fig. 5B1,B2 are in arbitrary units and intensities cannot be directly compared between the aboral body wall (Fig. 5B1) and the ambulacral (Fig. 5B2) samples because signal intensity is affected by sample thickness. The large ovoid regions without distinguishable collagen diffraction spectra correspond to voids in aboral body wall skeleton and collagenous tissue that are overlain on the body wall surface by papulae (thin‐walled finger‐like protrusions that mediate gas exchange in starfish) and pedicellariae (pincer‐like organs that remove debris and encrusting organisms from the body wall surface), as seen Fig. 1D. In the ambulacrum, collagen spectra are detectable at scan points along the midline between the antimeric ambulacral ossicles (arrowheads in Fig. 4B1), which corresponds with the position of interossicular ligaments that link the ambulacral ossicles (Fig. 3D–F).

A map of the ratio of diffraction intensity from collagen to the mineral scattering intensity (I_q5col_/I_q5min_ Fig. 5C1,C2) shows that the background scattering from the ossicles is proportionally higher in the ambulacrum than in the aboral body wall (note the different scales). This can be attributed to thicker ossicles in the ambulacrum and/or proportionally higher amounts of collagen in the aboral body wall.

### SAXD reveals collagen fibril orientation and differences in the degree of fibril orientation in starfish aboral body wall and ambulacrum

Measurement of the azimuthal intensity distribution of the 5th order reflection I_5_(χ) enabled determination of collagen fibril orientation in body wall samples. Thus, Fig. 5D1,D2 show vector plots overlaid on grayscale X‐ray transmission maps, with the orientation of vectors representing mean collagen fibril orientation at each scan point. In the aboral body wall the carinal ossicles are surrounded by fibrils aligned with the longitudinal axis of the starfish ray (black arrow). Fibrils adjacent to the reticular ossicles are orientated towards the voids in the ossicular lattice (white arrows), perpendicular to the ossicle surface.

In addition to fibril orientation, the extent to which fibrils are parallel (called degree of orientation here) can be determined from the inverse of the width of the azimuthal integrated intensity profile $\left(\frac{1}{\Delta \chi_{0}}\right)$. Azimuthally wide diffraction peaks (large Δχ_0_ > 10°) indicate high variation in the angular orientation of fibres. The scaled (from 0 to 1, normalized using a standard minimum and maximum for all the samples) mean degree of orientation for aboral body wall samples (0.13, SD 0.20) was twice that of ambulacrum samples (0.06, SD 0.03). These correspond to azimuthal diffraction peaks with mean FWHM of ~ 17° and ~ 35°, respectively. The degree of variation at different scan points is illustrated by the lengths of the vectors in Fig. 5D,D2. The longer the vector, the more parallel are the fibres at each scan point. The vector length varies more in the aboral body wall (the standard deviation of the vector length in the aboral samples is nearly seven times that of ambulacral samples), i.e. the variation in degree of orientation was higher in the aboral body wall samples than in the ambulacrum.

### D‐period length and O/D ratio of collagen fibrils differ between starfish aboral body wall and ambulacrum

**Figure 6:**
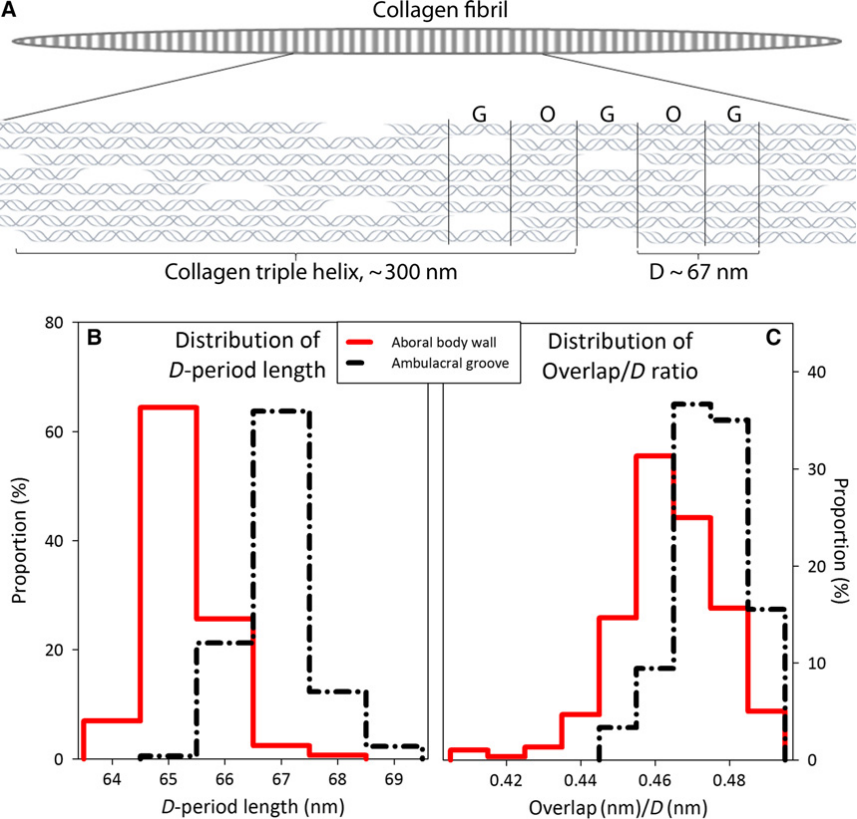
*Asterias rubens* body wall collagen fibril D‐period length and O/D ratio. (A) Simplified diagram of collagen triple helical molecule arrangement within a collagen fibril. The striated collagen fibrils are formed of triple helical collagen molecules that are arranged in a staggered manner leading to electron dense and light regions (overlap, O; gap, G). The combined length of one overlap and gap region equals a D‐period (D). The length of the D‐period and O/D ratio can be used as a measure to characterize collagen fibrils. (B) The histogram shows the distribution of D‐period length in aboral body wall (red solid line) and ambulacral groove (black dotted line) divided in bins of D < 64.5 nm; 64.5 nm ≤ D < 65.5 nm, 65.5 nm ≤ D < 66.5 nm, 66.5 nm ≤ D < 67.5 nm, 67.5 nm ≤ D < 68.5 nm and 68.5 nm ≤ D < 69.5 nm. Aboral body wall collagen fibrils have a lower mean D‐period than ambulacral ones. (C) The histogram shows the distribution of O/D ratios in aboral body wall (red solid line) and ambulacral groove (black dotted line) divided in bins with 0.01 ratio intervals. A lower O/D ratio is observed for the aboral body wall relative to the ambulacrum.

Collagen fibril D‐period lengths (see Fig. 6A, which shows a diagram of collagen fibril organization) calculated using the 5th order meridional Bragg peak position q_5_, for each scanning point with collagen intensity above the threshold revealed a significant difference in D‐period length between the aboral body wall and ambulacrum (*P* < 0.001; two‐tailed *t*‐test assuming equal variance) (Fig. 6B, D‐period distribution). The mean D‐period for scanning points in aboral body wall was 65.27 nm (SD 0.60, the combined number of scan points from the two aboral body wall samples included in the analysis; *n* = 504) and in the ambulacrum was 66.95 nm (SD 0.62, *n* = 269). The D‐period length was also more variable within each scan point in the ambulacral groove than in the aboral body wall. The range of D‐values for each scan point was calculated as described in the methods (*w*_*m(aboral)*_ = 0.012 nm^−1^, *w*_*m(ambulacrum)*_ = 0.015 nm^−1^, *w*_*crys(chicken)*_ = 0.011 nm^−1^) and the mean ranges were D ± 0.72 nm for ambulacrum and D ± 0.36 nm for the aboral body wall, again a possible indication of X‐rays penetrating through multiple collagenous layers in the ambulacrum with differential D‐period lengths.

The O/D ratios calculated using the ratio of I_8_/I_5_ (by numerically solving the following equation for O/D $\frac{I_{8}}{I_{5}}={\left(\frac{5}{8}\right)}^{2}\left(\frac{sin\left(\pi 8\frac{O}{D}\right)}{sin\left(\pi 5\frac{O}{D}\right)}\right))$ were also significantly different between the two regions of the body wall (*P* < 0.001; two‐tailed *t*‐test assuming equal variance; Fig. 6C, O/D distribution). One limitation with utilizing the step function and experimentally measured intensity ratios to estimate O/D is the lack of supporting structural data on echinoderm collagens. Therefore, when using the above‐mentioned equation to solve O/D it was assumed that electron densities within overlap and gap regions were constant and that the starfish collagen O/D ratio would be 0.4–0.5, i.e. similar to reported O/D values for mammalian collagen fibrils (Sasaki & Odajima, 1996a), as to the best of our knowledge there have been no reported studies of echinoderm collagen fibril organization being radically different from mammalian fibrils (see Discussion for more details). The O/D ratio was lower in the aboral body wall (0.468, SD 0.015) than in the ambulacrum samples (0.479, SD 0.009). If the differences in D‐period length and O/D ratio are both taken into account, the average lengths of overlap and gap regions in the aboral body wall are 30.55 and 34.72 nm, respectively (O+G=D, therefore O=0.468×D and $G=\left(1-O\right)\times D$). Average O and G lengths for the ambulacrum were 32.07 and 34.88 nm, respectively, showing that with a longer D‐period, the length of the overlap region increases, whereas the gap length remains more or less constant.

## Discussion

We report here a detailed analysis of body wall structure in the starfish *A. rubens*, employing histological, micro‐CT and SAXD techniques. Use of these complementary approaches has provided new insights into the functional anatomy of the starfish body wall, as discussed below.

### Trichrome staining reveals the anatomy of body wall interossicular muscles and ligaments in *A. rubens*

Trichrome staining of sectioned starfish has been employed previously for analysis of the structure and composition of the body wall, revealing that it is a composite of calcite ossicles linked by interossicular muscles and ligaments (O'Neill, 1989; Wilkie et al. 1995; Wilkie, 2001; Motokawa, 2011; Ben Khadra et al. 2015). The application of this histological staining technique to the common European starfish *A. rubens* adds to these body of data. In particular, high magnification photographs show how interossicular ligaments and muscles insert onto adjacent ossicles, with collagen fibrils and muscle fibres, respectively, wrapping around struts near to the ossicle surface (Fig. 4D'), findings that are consistent with a previous study on the longitudinal interambulacral muscles of the starfish *Pycnopodia helianthoides* Brandt, 1835 (Forcipulatida) (Wilkie et al. 1995). No intermediate tendons linking muscles with ossicles were observed. Thus, cavities in the calcite stereom near to the surface of the ossicle contain the loop‐shaped straps of both muscle fibres and collagen fibrils (Fig. 3A,B). This anatomical arrangement provides multiple sites of interaction between the soft (muscle and collagen) and hard (ossicle) tissue of the starfish body wall and may enable stiffened collagenous ligaments to bear loads when starfish are static and inbetween changes in posture initiated by muscle contraction.

Analysis of the structure of the aboral body wall in the starfish *E. spinulosus* (O'Neill, 1989) revealed that it has an ossicular layer consisting of ossicles and collagenous tissue located between inner and outer collagenous dermal layers, muscle and epithelium, with the collagenous tissue thickest in the inner dermal layer. The organization of collagen fibrils in the ossicular layer resembles an orthogonal web where the bulk of the collagen fibrils surround the ossicles but do not insert directly into them. This contrasts with the body wall of *A. rubens*, where collagenous tissue is closely associated with the ossicles and many fibrils penetrate into the ossicles to form loop‐shaped straps around calcite struts, as discussed above. More recently, Motokawa (2011) analysed the structure of the body wall in the starfish *Linckia laevigata* Linnaeus, 1758 (Valvatida), revealing that in this species the aboral body wall has an exceptionally thick inner dermal collagenous layer. By way of comparison, in *A. rubens* the aboral body wall ossicles are evenly surrounded by collagenous tissue without thickening of the inner dermis (Figs 2 and 3; Wilkie, 2001). The presence of a thickened inner dermal collagenous layer in *E. spinulosus* and *L*. *laevigata* accounts for the increased stiffness and rigidity of the body wall in these species in comparison with *Asterias* (Eylers, 1976; Marrs et al. 2000; Motokawa, 2011). Differences in body wall structure in the Asteroidea have been discussed in detail previously with respect to phylogeny, evolutionary success, ecology and habitat, defence strategies, mode of feeding and reproductive behaviour (Blake, 1990; Gale, 2011; Mah & Blake, 2012; Feuda & Smith, 2015). For example, the mechanical properties of the body wall influence the flexibility of the rays, limiting and/or enabling specific feeding behaviour (Gale, 2013) and the thickened dermal tissue in the sturdy rays of tropical shallow water valvatids and spinulosids such as *L. laevigata* and *E. spinuslosis* has also been attributed to an adaptation against extensive predation (Blake, 1983).

### X‐ray microtomography reveals the 3D structure of body wall ossicles in *A. rubens*

To the best of our knowledge this is the first study to employ micro‐CT to analyse the 3D anatomy of the body wall ossicular skeleton of an extant starfish species. Use of this technique has generated high resolution images that are informative from a functional perspective. The shapes and interactions of the different ossicle types that form the ray body wall in *A. rubens* are revealed in unprecedented detail.

The lateral, reticular and carinal ossicles, which form the skeleton of the aboral body wall, form a mesh‐like structure with overlapping lozenge‐shaped ossicles organized in rings that surround voids in the body wall skeleton. This ‘loose’ arrangement of ossicles is of functional significance for gas exchange because the voids are overlaid by clusters of papulae (Figs 1 and 2), finger‐like extendable and retractable exvaginations of the coelomic lining of body wall. The external epithelium and coelomic epithelium of the papulae are only separated by thin layers of collagenous tissue and muscle and thus they provide a large surface area for gas exchange between the coelomic fluid and external seawater (Brusca et al. 2016). The mesh‐like organization of ossicles that form the aboral body wall skeleton is also of functional relevance for changes in body posture in *A. rubens*. Thus, the contractile state of small interossicular muscles that link the aboral body wall ossicles (Fig. 3A,B) will determine the shape and size of a ring of ossicles. This can be seen in Fig. 4C, where the shape and size of the voids in aboral skeleton are highly variable, reflecting the particular posture of the ray when the animal was fixed. Changes in the shape and posture of the rays are required for several types of starfish behaviour. For example, when starfish are upturned by strong water currents, they exhibit a righting response, during which the rays twist and/or bend to enable the tube feet on the oral side of the ray to gain contact with the substratum (Pollis & Gonor, 1975). Likewise, when starfish feed on prey such as mussels they adopt a humped posture, with the rays bent so that tube feet proximal to the mouth are able to attach to the valves of the mussel and tube feet in the distal region of the rays maintain attachment to the substratum (Norberg & Tedengren, 1995). A similar posture is adopted during spawning (gamete release), increasing the dissemination of gametes in the water column (Himmelman et al. 2008). Furthermore, flexion of rays is aided by contraction of the apical muscle, a midline thickening of the longitudinally orientated muscle beneath the coelomic epithelium (Figs 2 and 3C).

In comparison with the aboral ray skeleton, the ossicles that form the oral ambulacral region of the rays in *A. rubens* are very different in their shape, size and packing. Thus, the ambulacral ossicles are long, thin and tightly packed, with their bases inserting in a groove formed by longitudinally adjacent cuboid‐shaped adambulacral ossicles. This anatomy can be inferred from detailed analysis of serial sections of starfish rays (Fig. 3) and scanning electron micrographs of individual ossicles (Wilkie et al. 1995), but analysis of images generated from micro‐CT is much easier and quicker. Furthermore, the micro‐CT data complement histological data in providing 3D representations of ossicle shapes and the ‘gaps’ between adjacent ossicles. For example, it can be seen how the aboral ‘head’ region of each ambulacral ossicle is shaped (Fig. 4D) to accommodate the muscles and ligaments that link antimeric and longitudinally adjacent ossicles (Fig. 3D). Similarly, indentations in the shaft of the ambulacral ossicles (Fig. 4D) accommodate the fluid‐filled tubes that connect each tube foot podium with its associated bulb‐shaped ampulla (Fig. 2).

Both carinal and ambulacral ossicles are organized in an overlapping manner, with each distal ossicle overlapping the more proximal one inclining towards the central disk. This overlap enables the bending, extension and retraction of the whole ray when the apical muscle within the aboral body wall and the interossicular longitudinal muscles in the ambulacrum contract or relax.

Heddle (1967) described body wall structure in the starfish *Luidia ciliaris* Philippi, 1837 (Paxillosida) and *Astropecten irregularis* Pennant, 1777 (Paxillosida), paying attention to the organization of antagonistic pairs of muscles involved in postural changes during locomotion and burrowing of the animals. It was noted that these burrowing starfish have three pairs of antagonistic muscles involved in abduction and adduction of the normally A‐shaped ambulacral groove. Only two of these pairs can be observed in *A. rubens* – the transverse supra‐ and infra‐ambulacral muscles and inner and outer lateral muscles (Fig. 2). The lack of a third antagonistic muscle pair is explained by the lack of superambulacral ossicles, structures found only in burrowing starfish that are evolutionarily distantly related to the Forcipulatida (Heddle, 1967; Feuda & Smith, 2015). The superambulacral ossicles and associated muscles are involved in changing the ray shape from a walking posture to a posture suitable for digging substrate from underneath the animal using tube feet. *Asterias rubens* is a non‐burrowing starfish and only changes its ray shape to crawl into crevices if stranded on the beach at low tide to avoid exposure to air and predators. Contraction of supra‐ambulacral and outer lateral abductor muscles flatten the shape of the starfish ray, increasing the angle between antimeric ambulacral ossicles forming the ambulacral arch. Contraction of infra‐ambulacral and inner lateral adductor muscles induces a more rounded and tubular ray shape, bringing the rows of tube feet directly below the animal and enabling locomotion with only vertical stress on tube feet.

### SAXD reveals properties of collagen in the body wall of *A. rubens*

Use of trichrome staining revealed the location of collagen in sections of the body wall of *A. rubens*, as discussed above. However, there are limitations in this approach to analysis of collagenous tissue. First, only thin (~ 10 μm) slices of tissue are sampled in each section, so a 3D perspective requires complex and time‐consuming reconstructions. Secondly, tissue fixation and processing for histological analysis causes changes in tissue structure (e.g. shrinkage) (Howat & Wilson, 2014) that may affect interpretation of the structural organization of collagen. Both of these limitations are circumvented with the application of the SAXD method to unfixed starfish body wall strips. Furthermore, SAXD can reveal ultrastructural properties of the fibrils (degree of orientation, O/D ratio) that cannot be determined with light/electron microscopy‐based histological methods.

X‐ray absorption maps revealed the positions of spines above reticular ossicles and the shape of the ‘gaps’ between the ambulacral ossicles, aiding the interpretation of the collagen X‐ray diffraction data. The SAXD scans themselves revealed an inhomogeneous distribution of collagen within the aboral body wall. Areas lacking detectable amounts of collagen correspond to voids in the body wall skeleton that underlie clusters of papulae on the body wall surface (Figs 1 and 2). The amount of collagenous components in comparison with mineral (ossicular) components was found to be higher in the aboral body wall in comparison with ambulacrum, consistent with the differences in the thickness of the ossicles in the two locations. The 2D orientation of individual collagen fibrils can be observed in high magnification images of trichrome sections (Fig. 3E,F), but SAXD enables quantification of mean fibril orientation *in vivo* and the degree of orientation of collagen fibrils, enabling comparisons between different locations in the body wall. The degree of fibril orientation within the aboral body wall was highly variable between scan points (the standard deviation of the vector length in the aboral samples was nearly seven times that of ambulacral samples). This probably reflects the complex lattice‐like organization of the ossicles in the aboral body wall where some parts of the tissue are under tension (high degree of orientation) and some completely relaxed (dispersed fibril orientation). In contrast, the ambulacral groove fibrils had a uniformly low degree of orientation (the mean vector length; degree of orientation for ambulacral samples was half of the mean length in the aboral body wall; note the different scales in Fig. 5D1,D2). It might be expected that collagen linking the highly regularly organized ambulacral ossicles would have a high degree of orientation. However, the thickness of the sample and the multidirectional fibres and fibrils (Fig. 3E) with macrocrimp (Fig. 3F) connecting antimeric ambulacral ossicles probably explains the uniform mean orientation with low degree of orientation in the ambulacrum.

The reported D‐value ranges (65.3 ± 0.6 nm for the aboral body wall and 67.0 ± 0.6 nm for ambulacral) are similar to the range of D‐values reported for mammalian collagen fibrils (65–67 nm; Krauss et al. 2009; Yang et al. 2015; Sasaki & Odajima, 1996a) and sea cucumber and sea urchin fibrils reported by Trotter et al. (1994) (65.7 ± 0.5 nm), but they are higher and less variable than those reported by Ferrario et al. (2017) (62.7 ± 2.8 nm for sea urchin, 63 ± 4.7 nm for starfish and 66 ± 1.6 nm for sea cucumber) and Ribeiro et al. (2012b) (59.2 ± 6.2 nm for sea urchin). The variation in D‐period length within echinoderms could reflect the differences in techniques (TEM can only be performed on fixed tissue/extracted fibrils, SAXD, on the other hand, requires no pre‐treatment of tissue and/or differences between species (all the previous echinoderm studies referred to in this paper used TEM images to measure the D‐period). The O/D ratios reported here (0.468, SD 0.015 for aboral body wall and 0.479, SD 0.009 for ambulacrum) are the first to be determined in an echinoderm. Interestingly, these are higher than previously described O/D ratios of 0.42–0.46 (depending on collagen type) for mammalian fibrils (Sasaki & Odajima, 1996b; Antipova & Orgel, 2010); however, the functional significance of this difference remains to be determined.

Comparison of the internal structure of collagen fibrils in the ambulacrum and aboral body wall of *A. rubens* revealed a significantly higher D‐period length, variability of D at each scan point and O/D ratio in the ambulacrum. The trend is consistent across and between the samples from each body location and possibly reflects differential fibril composition at sites experiencing varying mechanical demands. At least three mechanisms explaining forms of intrafibrillar deformation in response to increasing tension have been described and recognized in the past (Sasaki & Odajima (1996a), see Fig. 6A for reference of fibril structure). Initially, extension of tissue parallel to the main fibril direction straightens crimped fibrils. Thereafter fibrils can deform by three mechanisms (i) homogeneous elongation of individual tropocollagen molecules (the length of D increases, O/D remains the same, refer to Fig. 6A); (ii) molecular slippage, where only the gap between longitudinally adjacent molecules increases (no change in D, but decrease in O/D) and/or (iii) inhomogeneous elongation of the molecules (elongation of only gap or overlap regions). Additionally the length of D has been shown to vary between and within tissues even when they are not under tension (Fang et al. 2012).

Until now, the correlation between D and O/D has only been studied in the context of changes at a single tissue location during/before and after extension. This study focuses on two different locations with no external strain applied. Therefore, the observed differences in D‐values and O/D ratios may not solely or primarily represent differences in the state of tension in the tissue. The structure of fibrils is determined by constituent tropocollagen molecules (e.g. hetero‐ vs. homotrimeric and type of collagen alpha chains) (Orgel et al. 2006; Antipova & Orgel, 2010) and the length of D varies between mammalian collagenous tissues (Fang et al. 2012). The differences in the observed D and O/D values between aboral body wall and ambulacrum could therefore arise either from differential strain conditions or the molecular structure of the fibrils. Fibrillar collagen isolated from *Asterias amurensis* Lütken, 1871 (Forcipulatida) body wall consists of (α1)_2_α2 heterotrimers, similar to mammalian type I collagen (Kimura et al. 1993). That study, however, does not specify whether the collagen was isolated from whole starfish or from the aboral body wall only. Nor, to the best of our knowledge, have any amino acid sequences of *A. amurensis* or *A. rubens* α1 and α2 procollagen chains been determined. Hence we cannot at this stage make any statements about the difference (or not) between aboral and ambulacral collagen molecular structure. We also acknowledge that without structural knowledge of the starfish collagen molecules at the amino acid level, the method of calculating O/D ratios using experimentally measured intensity ratios has some limitations and the interpretation of the data may be reviewed in the future. Nevertheless, the O/D ratios determined here in *A. rubens* give an indication of innate differences between collagenous tissue in different body locations.

### Future directions

The present study provides detailed insights into ossicle shape and organization, the structural characteristics of collagenous tissue and the positions of muscles in the body wall of *A. rubens*. Determination of the anatomical organization of ossicles may provide a basis for experimental investigation of structure–function relationships. This study also highlights the lack of knowledge of *in vitro/vivo* ultrastructural properties of collagen fibrils from non‐mammalian animals. Comparative studies between tissues within species and between animals from different clades are required to understand mechanisms such as collagenous tissue mutability. Our study is a first step in this direction.

Further studies using *in situ* micromechanical testing during SAXD scanning could bring insights into mechanisms of intrafibrillar deformation and how mechanisms of collagenous tissue mutability work in concert with spatial gradients in collagen fibril composition/re‐orientation and the interconnecting ossicular network leading to large‐scale mechanical and postural changes in starfish. Using the combination of micromechanical testing and SAXD, we have recently shown that the mechanism of collagen mutability in sea cucumbers involves changes in interfibrillar stiffening rather than in any intrinsic changes in the properties of the collagen fibrils themselves (Mo et al. 2016). In inhomogeneous ossicle‐tissue networks such as the starfish body wall, changes in MCT properties could mechanically affect the links to and between the ossicle network, leading to flexion, torsion, etc. Changes in fibril strain and orientation during *in vitro* micromechanical testing can be tracked via changes in the SAXD patterns reflective of four different mechanisms for collagenous tissue elongation: (i) straightening of the fibrillar crimps; (ii) fibril reorientation; (iii) interfibrillar displacement (changes in the interfibrillar cohesion) and (iv) stretching of the fibrils themselves (Sasaki & Odajima, 1996a; Gupta, 2008).

Combined information on the material properties, shapes and organization of the ossicles and known material properties of the collagenous and muscle components could also be utilized to build a model of starfish arm movement. The model would provide information about the unique mode of locomotion and postural behaviour of starfish. In addition, the model could be used to simulate how the mechanics of composite materials can be modulated through changing the architecture, to make them softer/stiffer and more/less extensible by changing the fibril to mineral component ratio and/or internal structure and spatial organization of one or more of the elements (e.g. the amount of collagen linking the ossicles or the orientation of the fibrils).

## Conclusion

This study provides a detailed description of body wall structure in the starfish *A. rubens*, illustrating the value of a multi‐technique approach when characterizing tissue composites. The data obtained provides a structural basis for investigation of the mechanical properties of the starfish body wall both *in vitro* and *in silico,* using *A. rubens* as a model organism.

## Author contributions

Concept/design, acquisition of data, data analysis/interpretation, drafting of the manuscript: L.M.B., H.S.G., M.R.E. Acquisition of data, data analysis: M.E., Y.L., G.R.D., N.J.T.; all authors contributed to revising the manuscript and approved the manuscript.
